# The Sustained Increase of Plasma Fibrinogen During Ischemic Stroke Predicts Worse Outcome Independently of Baseline Fibrinogen Level

**DOI:** 10.1007/s10753-014-9838-9

**Published:** 2014-02-16

**Authors:** Marta Swarowska, Aleksandra Janowska, Agnieszka Polczak, Aleksandra Klimkowicz-Mrowiec, Joanna Pera, Agnieszka Slowik, Tomasz Dziedzic

**Affiliations:** Department of Neurology, Jagiellonian University Medical College, ul. Botaniczna 3, 31-503 Kraków, Poland

**Keywords:** fibrinogen, inflammation, outcome, stroke, brain infarction

## Abstract

Hyperfibrinogenemia at the beginning of ischemic stroke is associated with poor outcome. We hypothesized that the sustained increase of plasma fibrinogen during stroke predicts outcome independently of baseline fibrinogen concentration. We included 266 patients with first-ever ischemic stroke in whom plasma fibrinogen level was measured on days 1, 7, and 14. The sustained fibrinogen’s increase was defined as the persistent elevation of fibrinogen’s concentration on days 7 and 14 by at least 20 % compared to the level on day 1. The functional outcome on day 30 was assessed using modified Rankin Scale (mRS). Favorable outcome was defined as mRS 0–1. The sustained increase of fibrinogen was found in 17 % of patients. On multivariate logistic regression analysis adjusted for age, NIHSS score, baseline fibrinogen >2.66 mmol/L, presence of infection, and hyperglycemia, the sustained fibrinogen’s level was associated with reduced chance of favorable outcome (OR: 0.17, 95 % CI: 0.06–0.48, *P* < 0.01).

## INTRODUCTION

Clinical studies have demonstrated that increased level of inflammatory parameters related to acute phase reaction such as interleukin-6 or C-reactive protein (CRP) predicts unfavorable outcome in ischemic stroke patients (reviewed in [1]). Fibrinogen belongs to acute phase proteins. Hyperfibrinogenemia in acute cerebral ischemia is associated with increased risk of death within 1 year after stroke [2] and poor functional outcome [3]. Elevated fibrinogen level predicts also unfavorable prognosis in stroke patients treated with intravenous thrombolysis [4, 5].

Cerebral ischemia triggers acute phase reaction, and the blood concentration of inflammatory parameters could rise during brain infarction [1]. There is, however, a significant interindividual variability in inflammatory response after stroke [6–8]. Most of the studies investigating the relationship between inflammatory parameters and stroke prognosis have used baseline and/or discharge values of studied markers and have not taken into account the kinetics of these parameters during stroke.

Two major groups of factors determine elevated levels of inflammatory parameters in stroke patients [9, 10]. The first group is related to pre-stroke conditions and includes genetic factors and comorbidities associated with subclinical inflammation (for example, diabetes mellitus, obesity, and atherosclerosis). These factors determine the level of inflammatory parameters at the beginning of stroke and reflect mainly chronic pre-existing inflammation. The second group includes factors related to acute phase reaction. These factors are responsible for inflammatory parameters’ increase during stroke and reflect acute inflammation. The impact of these two groups of factors on stroke outcome could be different. We hypothesized that the *sustained increase* of plasma fibrinogen during stroke, even in patients with low baseline fibrinogen concentration, predicts outcome independently of baseline fibrinogen concentration.

## METHODS

We retrospectively analyzed the prospectively collected data on prognosis in stroke patients. The consecutive patients with first-ever ischemic stroke admitted to our stroke unit within 24 h after stroke onset were eligible for the study. The patients were recruited to the study between January 2011 and September 2012. The only exclusion criterion was the lack of patient’s consent for participation in the study. No patient was treated with intravenous thrombolysis.

All patients underwent head CT scan within 24 h after stroke onset. Stroke severity on admission was assessed using National Institute of Health Stroke Scale (NIHSS).

Arterial hypertension was diagnosed when its presence was documented in medical records or when at least two readings of blood pressure were ≥140 mmHg (systolic) and ≥90 mmHg (diastolic) after the acute phase of stroke. The diagnosis of diabetes mellitus was made when (1) the patient had the recognized diabetes mellitus before stroke as written in medical records and/or took hypoglycemic drugs before stroke; (2) fasting plasma glucose measured on days 6–10 was ≥7.0 mmol/L or fasting plasma glucose was 6.1–6.9 mmol/L and 2-h plasma glucose was ≥11.1 mmol/L after oral glucose tolerance test. A patient was defined as a smoker if there was a history of cigarette smoking during the last 5 years.

Plasma fibrinogen level was determined on days 1, 7, and 14 using modified Clauss method (Dade Behring, Marburg, Germany). Hyperfibrinogenemia was defined as plasma concentration >3.5 g/L. We defined arbitrarily the sustained increase of plasma fibrinogen as persistent elevation of fibrinogen’s concentration on days 7 and 14 by at least 20 % compared to the level on day 1. In addition, the fibrinogen’s increase by at least 30 % was considered in statistical analysis.

The functional outcome at day 30 was determined using modified Rankin Scale (mRS). The favorable outcome was defined as mRS 0–1.

The study protocol was approved by the Bioethics Committee of Jagiellonian University, and each participant gave the informed consent.

The *χ* ^2^ test was used to compare proportions and Mann–Whitney’s test to compare continuous variables between groups. Friedman’s ANOVA was used to compare serial fibrinogen measurements. Values of less than 0.05 were considerate to indicate statistical significance. Logistic regression analysis was used to assess if the sustained increase of fibrinogen is an independent predictor of favorable outcome. Since the baseline (day 1) fibrinogen level did not fulfil the linearity assumption of an interval-dependent variable, we divided the patients into two groups according to the median value: those with low (≤2.66 mmol/L) and those with high baseline fibrinogen (>2.66 mmol/L). Logistic regression was also used to assess the independent correlates of the sustained increase of fibrinogen. The variables with *P* < 0.10 on univariate analysis were included in multivariate analysis. The calculations were performed using the program STATISTICA for Windows (version 10, Statsoft, Poland).

## RESULTS

Three hundred thirty patients fulfilled the inclusion criteria. Sixty one (18 %) of them was lost, because they died within 14 days after stroke onset, did not agree to participate, or there was a lack of important data (outcome, fibrinogen measurements). The final cohort included 266 patients (median age [IQ]: 70.0 [64–78], 47.7 % men). The lost patients did not differ significantly from the final cohort in terms of age (median [IQ]: 75 [65–83] vs 70 [64–78], *P* = 0.06) and NIHSS score on admission (median [IQ]: 13 [8–21] vs 11.5 [8–16], *P* = 0.17); however, the lost patients had the higher plasma fibrinogen on day 1 (median [IQ]: 2.9 [2.6–3.4] vs 2.7 [2.3–3.1], *P* < 0.01).

**Fig. 1:**
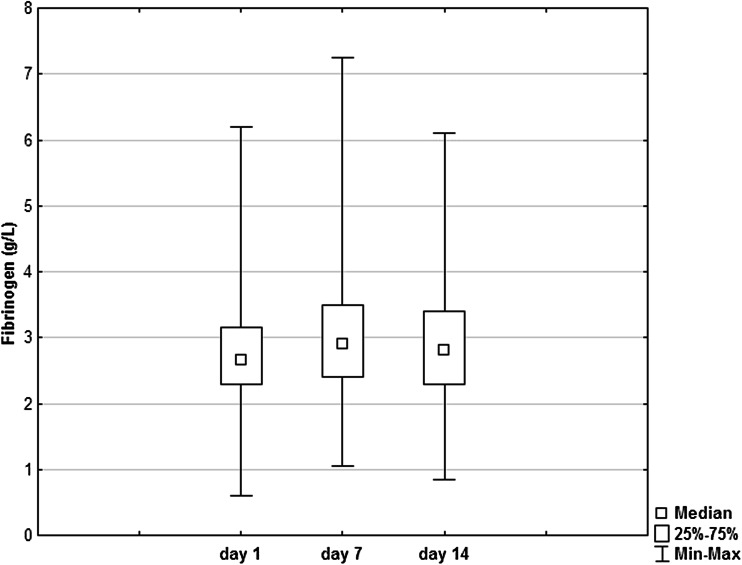
Plasma fibrinogen’s concentration during stroke. No significant change in fibrinogen’s concentration was found during observation period (*P* = 0.13, non-parametric ANOVA, df = 2).

The plasma fibrinogen levels are shown on Fig. 1. Friedman’s ANOVA for the three groups (df = 2) did not reveal any significant difference (*P* = 0.13). Wilcoxon’s signed-rank test (without Bonferroni correction) demonstrated the difference between fibrinogen on day 1 and fibrinogen on day 7 (*P* = 0.03).

**Fig. 2:**
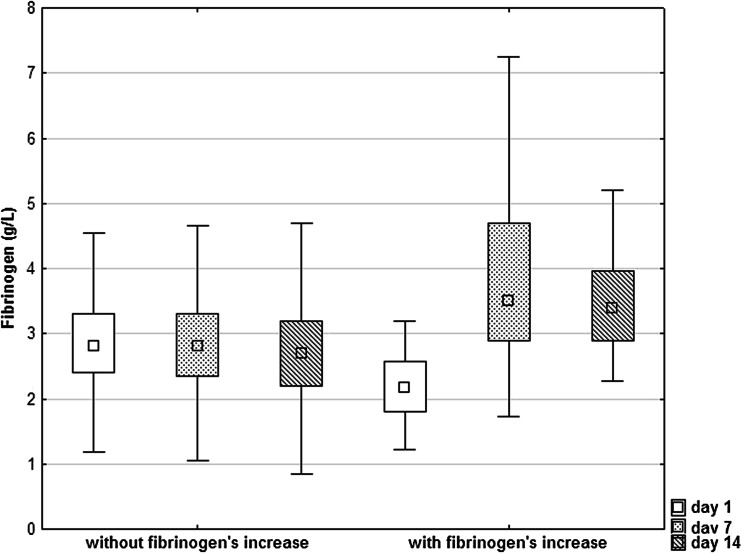
Plasma fibrinogen’s concentration in patients with and in those without sustained fibrinogen’s increase. The data are shown as a median with interquartiles (the box) accompanied by the minimal and maximal values.

The sustained increase of fibrinogen (e.g., fibrinogen concentrations on days 7 and 14 higher by 20 % compared to fibrinogen on day 1) was found in 45 patients (16.9 %). The fibrinogen’s concentrations in patients with sustained fibrinogen’s increase and patients without it are shown in Fig. 2. Table 1 shows the characteristics of patients with sustained fibrinogen’s increase and those without such an increase. The patients with sustained fibrinogen’s increase less often suffered from atrial fibrillation, had higher plasma glucose on admission and triglyceride level, and lower fibrinogen concentration on day 1. They also more frequently had in-hospital pneumonia.

**Table 1:** Characteristics of Stroke Patients with Sustained Fibrinogen’s Increase and those Without Sustained Fibrinogen’s Increase

	Patients with sustained fibrinogen’s increase (*N* = 45)	Patients without sustained fibrinogen’s increase (*N* = 221)	*P*
Age, median (IQ)	68 (62–74)	71 (64–79)	0.09
Men, n (%)	23 (51.1)	104 (47.1)	0.62
Hypertension, n (%)	28 (62.2)	148 (67.0)	0.54
Diabetes mellitus, n (%)	13 (28.9)	44 (19.9)	0.18
Previous myocardial infarction, n (%)	3 (6.7)	23 (10.4)	0.44
Atrial fibrillation, n (%)	2 (4.4)	39 (17.6)	0.02
Smoking, n (%)	13 (28.9)	60 (27.1)	0.81
TIA prior to stroke, n (%)	5 (11.1)	16 (7.2)	0.38
NIHSS score on admission, median (IQ)	13 (10–18.5)	11 (8–16)	0.06
Systolic blood pressure on admission (mmHg), median (IQ)	160 (150–170)	160 (140–180)	0.42
Diastolic blood pressure on admission (mmHg), median (IQ)	90 (90–100)	90 (80–100)	0.86
Glucose on admission (mmol/L), median (IQ)	6.9 (5.8–9.4)	6.5 (5.4–7.8)	0.04
Fasting glucose (mmol/L), median (IQ)	6.0 (5.4–7.0)	5.9 (5.1–7.1)	0.40
Total cholesterol (mmol/L), median (IQ)	5.7 (4.6–6.7)	5.2 (4.5–6.3)	0.12
LDL-cholesterol (mmol/L), median (IQ)	3.5 (2.9–4.8)	3.4 (2.6–4.2)	0.32
HDL-cholesterol (mmol/L), median (IQ)	1.2 (1.1–1.6)	1.3 (1.1–1.5)	0.66
Triglyceride, (mmol/L), median (IQ)	1.5 (1.3–2.2)	1.3 (1.1–1.7)	<0.01
WBC count (/μL), median (IQ)	8300 (6200–10,400)	8200 (6900–10,700)	0.83
Fibrinogen—day 1 (g/L), median (IQ)	2.2 (1.8–2.6)	2.8 (2.4–3.3)	<0.01
Fibrinogen—day 7 (g/L), median (IQ)	3.5 (2.9–4.7)	2.8 (2.3–3.3)	<0.01
Fibrinogen—day 14 (g/L), median (IQ)	3.4 (2.9–4.0)	2.7 (2.2–3.2)	<0.01
Hyperfibrinogenemia on day 1, n (%)	1 (2.2)	41 (18.5)	0.01
Hyperfibrinogenemia on day 7, n (%)	21 (46.7)	42 (19.0)	<0.01
Hyperfibrinogenemia on day 14, n (%)	19 (42.2)	36 (16.3)	<0.01
In-hospital pneumonia, n (%)	10 (22.2)	25 (11.3)	0.048
Urinary tract infections, n (%)	14 (31.1)	94 (42.5)	0.15
mRS 0–1 at day 30, n (%)	12 (26.7)	103 (46.6)	0.01

*IQ* interquartiles, *mRS* modified Rankin Scale, *NIHSS* National Institute of Health Stroke Scale, *TIA* transient ischemic attack, *WBC* white blood cells

Multivariate logistic regression analysis including variables that reached *P* < 0.1 on univariate analysis (age, NIHSS score, atrial fibrillation, plasma glucose on admission, triglycerides, fibrinogen on day 1, pneumonia) showed that higher NIHSS score (OR: 1.09, 95 % CI: 1.02–1.16, *P* < 0.01), hypertriglyceridemia defined as triglyceride >2.3 mmol/L (OR: 3.32, 95 % CI: 1.17–9.39, *P* = 0.02), and baseline fibrinogen >2.66 mmol/L (OR: 0.07, 95 % CI: 0.02–0.19, *P* < 0.01) were independently associated with sustained fibrinogen’s increase during stroke.

On univariate analysis, the sustained fibrinogen’s increase ≥20% was associated with reduced chance of favorable outcome (OR: 0.41, 95 % CI: 0.20–0.85, *P* = 0.01). Other variables significantly associated with favorable outcome on univariate analysis were age (OR: 0.95, 95 % CI: 0.93–0.97, *P* < 0.01), NIHSS score on admission (OR: 0.75, 95 % CI: 0.69–0.80, *P* < 0.01), presence of infection including pneumonia and urinary tract infections during hospitalization (OR: 0.39, 95 % CI: 0.23–0.65, *P* < 0.01), hyperglycemia defined as fasting glycemia on day 1 >7.0 mmol/L (OR: 0.50, 95 % CI: 0.27–0.90, *P* = 0.02), and baseline fibrinogen >2.66 mmol/L (OR: 0.43, 95 % CI: 0.26–0.71, *P* < 0.01). On multivariate analysis, after adjustment for the abovementioned confounders, fibrinogen’s increase ≥20% remained an independent predictor of favorable outcome (OR: 0.17, 95 % CI: 0.06–0.48, *P* < 0.01). Other significant predictors of outcome were: age (OR: 0.95, 95 % CI: 0.91–0.98, *P* < 0.01), NIHSS score (OR: 0.77, 95 % CI: 0.71–0.83, *P* < 0.01), and baseline fibrinogen >2.66 mmol/L (OR: 0.22, 95 % CI: 0.10–0.49, *P* < 0.01). The similar results of multivariate analysis were obtained when fibrinogen’s increase ≥30 % was used in the model (OR: 0.14, 95 % CI: 0.05–0.43, *P* < 0.01).

## DISCUSSION

In this study, we hypothesized that not only baseline concentration of plasma fibrinogen but also its persistent elevation during stroke has the negative effect on outcome. We found that the sustained increase of fibrinogen level during stroke is associated with reduced chance of favorable outcome and independent baseline fibrinogen level.

The elevated level of inflammatory markers such as CRP or fibrinogen at the beginning of stroke may reflect the burden of atherosclerosis and/or the presence of concomitant risk factors (e.g., hypertension, diabetes mellitus, obesity) [9]. In addition, the blood level of these markers could rise during stroke as a part of the acute phase reaction [1]. There is a significant interindividual variability in inflammatory response after stroke. In one study where serum CRP was measured within 24 h after stroke onset, within 48–72 h and at hospital discharge, persistently normal values were seen in 19.5 % of patients, increasing values in 6.3 %, decreasing values in 28.1 %, and persistent elevation in 46.1 % [6]. In another study, 38 % of ischemic stroke patients had normal CRP levels on days 1 and 90, 40 % had elevated CRP on both days, 14 % demonstrated an increase from normal to elevated, and 8 % showed a decrease from elevated to normal values [7]. In that study, in 28 % of patients, fibrinogen level did not change by >50 mg/dL between days 1 and 90, 59 % of patients showed an increase, and 13 % showed a decrease between both time points.

Although in our cohort plasma fibrinogen level rose during the stroke, non-parametric ANOVA (Friedman’s test) did not reveal the significant differences between fibrinogen concentration measured on days 1, 7, and 14. In some studies, fibrinogen level rose significantly during stroke course [7, 11]; whereas, in other studies, no evidence of time trend was seen [12] or fibrinogen concentration increased gradually during serial measurement, but did not reach significant level [3, 13].

The sustained elevation of fibrinogen level by 20 % was found in 17 % of our patients. Interestingly, only 2.2 % of them had hyperfibrinogenemia on day 1. Of note, low fibrinogen level on day 1 predicted the increase of this protein’s concentration during stroke. More severe stroke and higher triglyceride level were other predictors of sustained fibrinogen’s increase. Stroke severity is a major determinant of acute phase reaction after stroke [1], and hypertriglyceridemia is associated with systemic inflammation [14]. The higher frequency of hypertriglyceridemia in patients without atrial fibrillation compared to those with atrial fibrillation (13.9 % vs 2.4 %, *P* = 0.03) could explain the significant association between atrial fibrillation and sustained fibrinogen’s increase found on univariate but not on multivariate analysis.

The observational studies are not able to demonstrate causality between increased level of inflammatory markers including fibrinogen and poor outcome after stroke. The interventional study with ancrod, the defibrinogenating agent, showed that this drug starting within 6 h after ischemic stroke symptom onset did not improve the outcome despite the success to achieve rapid initial defibrogenation and avoid prolonged hypofibrinogenemia [15]. Although the lack of benefit after ancrod administration could be related to the side effects of the drug (increased incidence of infections, renal failure, and intracerebral bleeding), it may also suggest that elevated level of fibrinogen is only the biomarker of prognosis, but not the target for the treatment. If the elevation of fibrinogen level is only epiphenomenon without direct effect on outcome, it is important to recognize the mechanisms leading to the increase of inflammatory molecules in blood during stroke, because these mechanisms could also influence the outcome and be a potential therapeutic target. On the other hand, if increased fibrinogen level has a direct deleterious effect on cerebral ischemia, it should be taken into consideration that therapy that lowers its level should be not limited to the first hours after stroke onset, but it needs to be continued longer to prevent the increase of fibrinogen in patients who had normal fibrinogen level on admission.

In conclusion, the sustained increase of fibrinogen during stroke, also in patients with low fibrinogen level on day 1, is associated with reduced chance of favorable outcome.
